# Short-latency inhibition mitigates the relationship between conscious movement processing and overly cautious gait

**DOI:** 10.1093/ageing/afaa230

**Published:** 2020-11-18

**Authors:** Toby J Ellmers, Elmar C Kal, James K Richardson, William R Young

**Affiliations:** College of Health, Medicine and Life Sciences, Brunel University London, UK; Centre for Cognitive Neuroscience, Brunel University London, UK; College of Health, Medicine and Life Sciences, Brunel University London, UK; Centre for Cognitive Neuroscience, Brunel University London, UK; Department of Physical Medicine and Rehabilitation, University of Michigan, USA; School of Sport and Health Sciences, University of Exeter, UK; College of Health, Medicine and Life Sciences, Brunel University London, UK

**Keywords:** conscious movement processing, inhibition, reinvestment, cautious gait, older people

## Abstract

**Background:**

Overly cautious gait is common in older adults. This is characterised by excessively slow gait, shortened steps, broadened base of support and increased double limb support. The current study sought to (1) evaluate if overly cautious gait is associated with attempts to consciously process walking movements, and (2) explore whether an individual’s ability to rapidly inhibit a dominant motor response serves to mitigate this relationship.

**Methods:**

A total of 50 older adults walked at a self-selected pace on an instrumented walkway containing two raised wooden obstacles (height = 23 cm). Trait conscious movement processing was measured with the Movement-Specific Reinvestment Scale. Short-latency inhibitory function was assessed using a validated electronic go/no-go ruler catch protocol. We used linear regressions to explore the relationship between these variables and gait parameters indicative of overly cautious gait.

**Results:**

When controlling for general cognitive function (MoCA), and functional balance (Berg Balance Scale), the interaction between trait conscious movement processing and short-latency inhibition capacity significantly predicted gait velocity, step length and double limb support. Specifically, older adults with higher trait conscious movement processing and poorer inhibition were more likely to exhibit gait characteristics indicative of cautious gait (i.e. reduced velocity, shorter step lengths and increased double limb support). Neither conscious movement processing nor inhibition independently predicted gait performance.

**Conclusion:**

The combination of excessive movement processing tendencies and poor short-latency inhibitory capacity was associated with dysfunctional or ‘overly cautious’ gait. It is therefore plausible that improvement in either factor may lead to improved gait and reduced fall risk.

## Key Points

- During certain situations, older adults will seek to consciously process their walking movements.
- Conscious movement processing has been linked to maladaptive gait behaviours (specifically overly cautious gait) and fall risk.
- However, we show that older adults with good inhibition may be able to suppress such conscious processing—and associated gait adaptations.
- Clinicians should assess both conscious movement processing and inhibition, as these represent potential targets for therapy.

## Introduction

Gait disturbances are common in older adults, affecting around 35% of those aged over 70 [1]. One frequently reported gait abnormality is disproportionately ‘cautious’ gait (relative to actual physical function) [2]. This is characterised by excessively slow gait (compared to age-relevant norms), shortened steps, broadened base of support (i.e. widened step width) and increased double limb support (i.e. both feet planted on the floor). Such behaviours are linked to greater fall risk [3], potentially due to their association with gait instability [4]. Overly cautious gait can be categorised as a higher level gait disorder [5]; it cannot be primarily attributed to deficient sensory or motor systems, but rather neuropsychological factors. More specifically, cautious gait is thought to arise when individuals who are fearful of falling *consciously* process (i.e. monitor or control) their walking movements to reduce the likelihood of falling [5].

The relationship between fear of falling and increased conscious movement processing is well documented in older adults [6, 7]. Greater conscious movement processing has also been reported in older adults who have recently fallen [8]. Consciously processed walking movements are, by definition, less ‘automatic’ [9]: they require longer to initiate [9], need greater cognitive resources to plan and execute [9, 10], and the resulting movements are slower, less efficient (i.e. require increased muscular activation) and more variable [9, 11, 12]. However, researchers have also proposed that consciously processing locomotion may serve a functional benefit during certain scenarios (for example, when walking across a slippery surface) [7]. This implies that safe, effective gait may be characterised by the flexible integration of both automatic and consciously processed stepping movements. We therefore speculate that excessively cautious gait arises when an individual is unable to inhibit consciously processing stepping movements during situations which do not warrant such conscious modes of motor control.

Masters and Maxwell argued that the degree to which an individual consciously processes their movements should be considered a personality trait [13]. Supporting this assumption, Uiga and colleagues [14] described that older adults with a trait propensity for conscious movement processing displayed cautious gait behaviour, taking longer to plan stepping actions, despite ultimately exhibiting greater stepping errors. In contrast, however, Mak, Young and Wong [15] recently observed a lack of association between trait conscious movement processing and gait behaviour in older adults during level-ground walking. Although the reason for this discrepancy is unknown, we propose that an individual’s ability to inhibit consciously processed behavioural responses may be a crucial mediating factor. Indeed, inhibition is argued to reflect—among other things—one’s ability to suppress dominant behavioural responses that are inappropriate for current task demands [16]. Therefore, despite possessing a trait propensity to consciously process movement, we predict that certain older adults may be better able to inhibit such conscious processing from translating to overly cautious gait.

The current study evaluated if overly cautious gait is associated with a trait propensity to consciously process movement, and explored whether an individual’s ability to rapidly inhibit a dominant motor response serves to mitigate this relationship.

## Methods

### Participants

A power analysis determined that 49 participants would be required to detect a significant improvement in R^2^ (of 0.17) when adding a trait conscious processing by inhibition interaction term to a linear regression model with five predictors in total (α = 0.05, β = 0.80; including measures of cognitive function and functional balance, trait conscious movement processing, inhibition and their interaction).^1^

A total of 50 community-dwelling older adults (aged>60; males: 15/50; mean ± *SD* age: 74.36 ± 7.12) were recruited from local community groups. Participants were free from any neurological, cardiovascular or musculoskeletal impairment that prohibited them from walking 10 m without a walking aid. Participants were excluded if they demonstrated major cognitive impairment (Montreal Cognitive Assessment [MoCA] score < 18/30 [17]), or if they were currently prescribed anxiety or dizziness medication. All participants had normal or corrected-to-normal vision. Institutional ethical approval was obtained from the local ethics committee and the research was carried out in accordance with the principles laid down by the Declaration of Helsinki. All participants provided written informed consent prior to participation. Demographic information is reported in Table 1.

**Table 1:** Demographic and primary outcome data

	Mean (SD)^a^	Range
***Participant demographics***		
Age	74.36 (7.12)	61–86
Gender (females: males)	35: 15	
Height (cm)	165.42 (8.85)	143–192
Weight (kg)	71.38 (14.55)	44–116
Berg balance scale (0–56)	52.60 (3.14)	42–56
Timed up and go (s)	11.10 (3.34)	7.00–22.64
Grip strength (kg/f)	24.54 (6.12)	12.30–53.75
Montreal cognitive assessment (0–30)	26.50 (2.82)	20–30
Falls in previous year, no. of participants	15/50	
No. daily medications	2.67 (2.28)	0–10
***Gait performance outcomes***		
Gait velocity (cm/s)	90.88 (23.98)	28.90–132.70
Step length (cm)	59.43 (12.20)	25.25–82.32
Base of support (cm)	12.43 (4.37)	4.06–26.69
Double-limb support (% gait cycle)	21.54 (6.12)	13.55–45.05
***Regression predictors***		
Trait conscious movement processing^*^ (10–60)	23.64 (11.45)	10–54
Inhibition accuracy (%)	50.7 (22.87)	0–90

^a^Unless stated otherwise, variables are reported as the mean (and standard deviation) and range.

^*^Trait conscious movement processing was assessed via the Movement-Specific Reinvestment Scale

### Protocol

Participants first completed the MoCA, followed by assessments of functional balance (Berg Balance Scale [BBS] [18]), and both trait conscious movement processing and inhibition function (see below). We collected additional baseline demographic data, including Timed up and Go (s), grip strength (kg/f) and number of medications currently taken. Next, participants completed five walks along a 6-m automated GAITRite walkway (CIR Systems Inc., Havertown, PA) located in a quiet, well-lit laboratory. The walkway contained two wooden obstacles (obstacle height = 23 cm) that participants had to step over (obstacle 1 = 2.5 m from start of the GAITRite walkway, obstacle 2 = 2.5 m after obstacle 1). To allow for initial acceleration and terminal deceleration, start and stop points were marked on the floor 1.5-m outside of the start and end of the walkway capture area, respectively.

### Outcome measures

#### Gait performance

We calculated four variables associated with cautious gait: gait velocity (cm/s), step length (cm), medio-lateral base of support (cm) and double limb support (% of gait cycle). Variables were averaged across the five trials. As older adults will adapt their stepping behaviour at least six steps before reaching an obstacle [19], gait data were analysed throughout the whole trial.

#### Conscious movement processing

The Movement-Specific Reinvestment Scale (MSRS) [20] was used to measure participants’ trait propensity to consciously process their movements [14, 15]. The scale assesses the degree to which an individual monitors and controls movement. Items are rated on a 6-point Likert scale (1 = *strongly disagree*; 6 = *strongly agree*), and summed to produce an overall score [14, 15]. Scores range from 10 to 60, with higher scores reflecting a higher trait propensity to consciously process movement.

#### Short-latency inhibition

We used a patented, custom-built reaction device to assess short-latency inhibition (henceforth referred to as ‘ReacStick’). It consists of a 107 cm rigid, lightweight shaft affixed to an 11 × 6 × 2.5 cm ‘ spacer box’ housing a linear accelerator, timing circuit, microprocessor, battery, liquid crystal display, and two light emitting diodes at the top of the spacer box (see [21] for graphical representation of the device). Participants sat with their dominant forearm resting comfortably on a horizontal table surface approximately 75 cm above the ground (as in [21–23]). The forearm was maintained in position so that its ulnar surface contacted the table, and the hand was held beyond the table edge. The experimenter held the ReacStick with the spacer box between the participant’s thumb and fingers. The device is programmed such that the light-emitting diodes illuminate at the instant of release on 50% of trials (randomly selected). The examiner and participant were both blinded to whether the diodes would illuminate on any given trial. Participants were instructed to catch the device solely on those trials in which the lights illuminate, and to let the device drop and hit the ground on the trials in which the lights remained off. Verbal instructions emphasised response accuracy, not speed. Nonetheless, this task assesses short-latency inhibition, as task success demands that responses had to be made in the 400 ms before the stick hits the floor. Participants carried out six practice trials, which included at least two ‘light off’ trials to ascertain that they understood instructions, and then 20 data collection trials. The percentage of trials in which the participant successfully refrained from catching the ReacStick during ‘light off’ trials was the outcome of interest in the present research. This variable is termed ‘Off Accuracy’ [21]. Previous research has described good test–retest reliability for this variable [21], and the methods described reflect a standardised and validated testing protocol [22].

Note, prior to the inhibition trials, participants completed 12 ‘simple reaction time’ trials, where speed *was* emphasised. For these trials, the lights remained off and participants instead caught the falling stick as quickly as possible [21]. Completing the simple reaction time trials prior to the light on/off inhibition trials ensured that catching the falling stick was the dominant response. Consequently, successfully letting the falling stick hit the floor during ‘light off’ trials thus represents the rapid inhibition (<400 ms) of a dominant response.

**Table 2:** Hierarchical Regression Models with **conscious movement processing** (MSRS) and **inhibition** (ReacStick ‘off’ accuracy) as predictors of gait performance, when controlling for functional balance and cognitive function.

**MODEL 1**					
Dependent variable: **Gait velocity**					
	*B* (*SE*)	[95% CI]	*P*	*R* ^2^	*R* ^2^ change
**Step 1**				.365 (***P* < 0.001**)	
Constant	.017 (.116)	[−.217, 0.250]	.888		
Functional balance (BBS)	.574 (.122)	[.327, 0.820]	**<.001**		
Cognitive function (MoCA)	.088 (.121)	[−.155, 0.332]	.469		
**Step 2**				.402 (***P* < 0.001**)	.036 (*P* = 0.266)
Constant	.012 (.116)	[−.221, 0.245]	.919		
Functional balance (BBS)	.568 (.131)	[.304, 0.833]	**<.001**		
Cognitive function (MoCA)	−.003 (.135)	[−.276, 0.269]	.981		
Conscious movement processing (MSRS)	.079 (.126)	[−.175, 0.332]	.536		
Inhibition (ReacStick ‘off’ accuracy)	.216 (.138)	[−.061, 0.493]	.124		
**Step 3**				.459 **(*P* < 0.001)**	.058 **(*****P*** = **0.036)**
Constant	.052 (.113)	[−.175, 0.279]	.648		
Functional balance (BBS)	.571 (.126)	[.317, 0.825]	**<.001**		
Cognitive function (MoCA)	−.046 (.131)	[−.311, 0.219]	.729		
Conscious movement processing (MSRS)	.184 (.130)	[−.079, 0.447]	.166		
Inhibition (ReacStick ‘off’ accuracy)	.213 (.132)	[−.053, 0.480]	.114		
Conscious movement processing by Inhibition	.231 (.107)	[.016, 0.446]	**.036**		
**MODEL 2**					
Dependent variable: **Step length**					
	*B* (*SE*)	[95% CI]	*P*	*R* ^2^	*R* ^2^ change
**Step 1**				.370 (***P* < 0.001**)	
Constant	.011 (.113)	[−.217, 0.239]	.924		
Functional balance (BBS)	.546 (.119)	[.306, 0.786]	**<.001**		
Cognitive function (MoCA)	.127 (.118)	[−.111, 0.364]	.288		
**Step 2**				.413 (***P* < 0.001**)	.043 (*P* = 0.204)
Constant	.003 (.112)	[−.223, 0.228]	.980		
Functional balance (BBS)	.570 (.127)	[.314, 0.826]	**<.001**		
Cognitive function (MoCA)	.051 (.131)	[−.212, 0.315]	.696		
Conscious movement processing (MSRS)	.148 (.122)	[−.097, 0.394]	.230		
Inhibition (ReacStick ‘off’ accuracy)	.191 (.133)	[−.078, 0.459]	.160		
**Step 3**				.469 **(*P* < 0.001)**	.055 **(*****P*** = **0.038)**
Constant	.041 (.109)	[−.179, 0.261]	.708		
Functional balance (BBS)	.572 (.122)	[.326, 0.818]	**<.001**		
Cognitive function (MoCA)	.011 (.127)	[−.246, 0.267]	.934		
Conscious movement processing (MSRS)	.249 (.126)	[−.006, 0.504]	.055		
Inhibition (ReacStick ‘off’ accuracy)	.188 (.128)	[−.070, 0.447]	.149		
Conscious movement processing by Inhibition	.221 (.103)	[.013, 0.430]	**.038**		
**MODEL 3**					
Dependent variable: **Base of support**					
	*B* (*SE*)	[95% CI]	*P*	*R* ^2^	*R* ^2^ change
**Step 1**				.315 (***P* < 0.001**)	
Constant	.014 (.123)	[−.232, 0.261]	.907		
Functional balance (BBS)	−.561 (.129)	[−.820, −.301]	**<.001**		
Cognitive function (MoCA)	−.034 (.128)	[−.291, 0.222]	.788		
**Step 2**				.317 (***P*** **=** **0.002**)	.002 (*P* = 0.932)
Constant	.015 (.125)	[−.237, 0.268]	.904		
Functional balance (BBS)	−.556 (.142)	[−.842, −.270]	**<.001**		
Cognitive function (MoCA)	−.010 (.147)	[−.306, 0.285]	.945		
Conscious movement processing (MSRS)	−.011 (.137)	[−.287, 0.264]	.934		
Inhibition (ReacStick ‘off’ accuracy)	−.056 (.149)	[−.356, 0.245]	.711		
**Step 3**				.318 **(*****P*** = **0.004)**	.001 (*P* = 0.808)
Constant	.010 (.128)	[−.249, 0.269]	.938		
Functional balance (BBS)	−.556 (.144)	[−.846, −.267]	**<.001**		
Cognitive function (MoCA)	−.005 (.150)	[−.307, 0.297]	.975		
Conscious movement processing (MSRS)	−.025 (.149)	[−.325, 0.275]	.868		
Inhibition (ReacStick ‘off’ accuracy)	−.055 (.151)	[−.359, 0.249]	.716		
Conscious movement processing by Inhibition	−.030 (.122)	[−.275, 0.215]	.808		
**MODEL 4**					
Dependent variable: **Double limb support**					
	*B* (*SE*)	[95% CI]	*P*	*R* ^2^	*R* ^2^ change
**Step 1**				.255 (***P*** = **0.001**)	
Constant	.007 (.128)	[−.251, 0.265]	.954		
Functional balance (BBS)	−.458 (.135)	[−.729, −.186]	**.001**		
Cognitive function (MoCA)	−.136 (.133)	[−.405, 0.132]	.312		
**Step 2**				.262 (***P*** = **0.007**)	.007 (*P* = 0.809)
Constant	.005 (.131)	[−.258, 0.268]	.970		
Functional balance (BBS)	−.425 (.148)	[−.723, −.126]	**.006**		
Cognitive function (MoCA)	−.098 (.153)	[−.406, 0.209]	.523		
Conscious movement processing (MSRS)	.057 (.142)	[−.230, 0.344]	.690		
Inhibition (ReacStick ‘off’ accuracy)	−.075 (.156)	[−.389, 0.238]	.630		
**Step 3**				.395 **(*P* < 0.001)**	.132 **(*****P*** = **0.003)**
Constant	−.057 (.121)	[−.301, 0.188]	.642		
Functional balance (BBS)	−.429 (.136)	[−.702, −.155]	**.003**		
Cognitive function (MoCA)	−.003 (.142)	[−.318, 0.253]	.819		
Conscious movement processing (MSRS)	−.105 (.141)	[−.388, 0.178]	.459		
Inhibition (ReacStick ‘off’ accuracy)	−.072 (.143)	[−.359, 0.216]	.617		
Conscious movement processing by Inhibition	−.356 (.115)	[−.587, −.124]	**.003**		

### Statistical analysis

We performed four hierarchical three-stepped moderation linear regression analyses (one regression per dependent gait variable), applying steps as recommended by Dawson [24]. Regressions were performed on standardised values. Control variables were entered in the first step. These were: functional balance (BBS) and general cognitive function (MoCA). In the second step, the predictor (conscious movement processing; MSRS) and moderator (inhibition; ReacStick ‘off’ accuracy) were entered. Finally, the interaction between the predictor and moderator (product term of standardised values) were added in the third step. The interaction terms were regarded to be relevant only if they significantly improved model fit (*R*^2^). As it is not advised to perform follow-up simple slope tests on variables without meaningful cut-off values (such as the predictor and moderator variables used in the present research), any significant interactions were instead plotted to aid interpretation [24]. As recommended [24], these slopes were plotted using values one standard deviation above/below the mean to reflect high/low MSRS and good/poor inhibition, respectively. For all regression analyses, the assumptions of homoscedasticity (by inspecting the standardised residuals by standardised predicted values plot), error-independence (Durbin–Watson values >1.62), lack of multicollinearity (variance inflation factors <1.4, tolerances >0.7, *r*s <0.51), and normal distribution of errors (as determined with Kolmogorov–Smirnov tests and inspection of histogram of residuals) were verified.

## Results

The mean and range of outcome (gait), predictor and moderator variables are described in Table 1. The hierarchical regression analyses are presented in Table 2.

**Figure 1:**
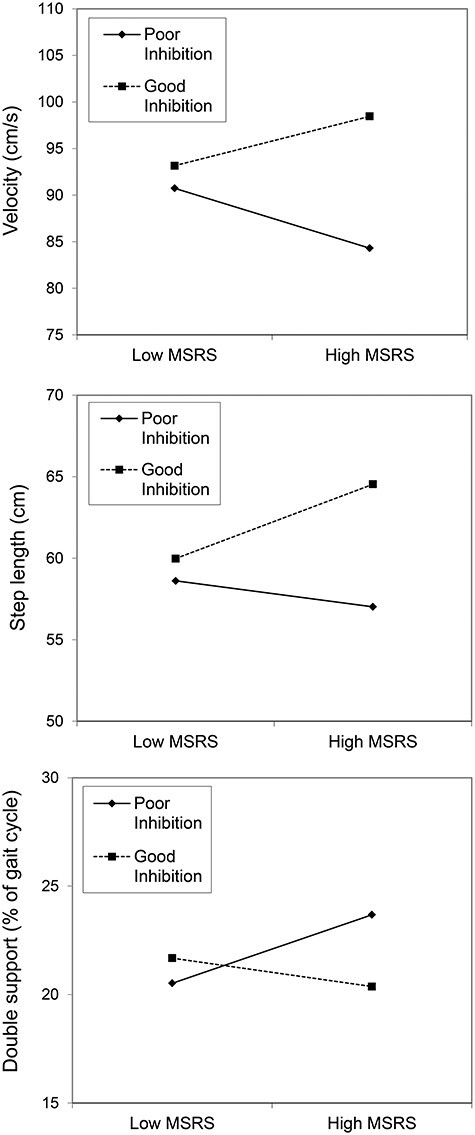
Interaction effects between conscious movement processing (MSRS) and inhibition (ReacStick ‘off’ accuracy) on gait velocity (top), step length (middle) and double limb support (bottom). Note, given the lack of validated cut-off points for high/low MSRS or good/poor inhibition, these slopes are instead plotted at one standard deviation above/below the mean.

The interaction terms (conscious movement processing by inhibition) significantly improved model fit for gait velocity, step length and double limb support, explaining an additional 5.8% (*P* = 0.036), 5.5% (*P* = 0.038) and 13.2% (*P* = 0.003) of variance, respectively. For each model, only the interaction term itself significantly predicted these gait variables—not conscious movement processing or inhibition independently (all *B*s between −0.075 and 0.216, *P*s > 0.124). The interaction effects are illustrated in Figure 1: Older adults with high trait conscious movement processing and poor inhibition were more likely to show gait performance indicative of overly cautious gait compared to older adults with high trait conscious movement processing and *good* inhibition (i.e. slower velocity, shorter step lengths and increased double limb support).

Neither MSRS nor inhibition significantly predicted base of support (all *B*s between −0.011 and − 0.056, *P*s > 0.934), and adding their interaction term did not significantly improve model fit either (*P* = 0.808; Table 2).

## Discussion

As predicted, the interaction between trait conscious movement processing and short-latency inhibition significantly predicted cautious gait behaviour in older adults (when controlling for functional balance and general cognitive function). That is, older adults with poor inhibition and strong conscious processing tendencies were more likely to exhibit overly cautious gait. This effect seemed most pronounced for double limb support (13.2% variation explained). The throw-and-catch model of human gait proposes that the trajectory and dynamics of a step are typically determined during the preceding dual stance phase [25]. Consciously processed walking movements, however, require longer to initiate [9]. As such, older adults with high trait conscious movement processing but poor inhibition may have prolonged their dual stance position to afford the time required to consciously plan and initiate the following step. As ReacStick protocol assesses short-latency inhibition (<400 ms), it is therefore possible that the mechanisms described above could occur on a cyclical (i.e. step-by-step) basis. This fits earlier work that showed more random, independent timing of stepping movements in cautious gait [4]—suggesting increased step-by-step control.

It is well accepted that fear of falling leads to increased conscious movement processing in older adults [6, 7]. Previous research has also highlighted clear links between fear of falling and cautious gait [4, 5]. While we did not directly assess fear of falling in the present research, our findings nonetheless suggest that (an inability to inhibit) conscious movement processing may—to some extent, at least—underpin overly cautious patterns of gait typically observed in fearful individuals [4, 5]. While these behaviours did not appear to directly impact safety in the present research (as no participant tripped on the obstacle), such overly cautious gait patterns are reliably linked to increased falls [3]. Consciously processed walking movements are not only slower and less efficient (as highlighted in the present research), they also require greater cognitive resources to plan and execute [9, 10]. We therefore suggest that an inability to suppress consciously processed (and overly cautious) gait is likely to directly impact safety in situations which do not provide the affordances (i.e. time or cognitive resources) required to carry out this mode of motor control.

Interestingly, neither trait conscious movement processing nor inhibition independently predicted gait behaviour. We propose that previous research reporting an absence of association between trait conscious movement processing and cautious gait in older adults (e.g. [15]) is likely due to differences in the ability to inhibit conscious intervention in movement. This suggests that an individual’s level of trait conscious processing may be insufficient for predicting gait outcomes when used in isolation, but may be relevant when used in combination with measurement of short-latency inhibition. In contrast, functional balance was a strong independent predictor of gait behaviour across all assessed variables (including base of support, which was not predicted by the interaction of conscious movement processing and inhibition). We interpret these findings to imply that deficits within functional balance are a primary cause of cautious gait in older adults. Such cautious behaviour may, therefore, largely reflect an adaptive response aimed at enhancing stability and safety. However, as the interaction between conscious movement processing and inhibition was also an independent predictor of gait behaviour, we propose that an inability to inhibit conscious processing may lead to *overly* cautious gait (i.e. disproportionate caution in relation to functional balance).

Experimentally induced conscious movement processing has been shown to result in cautious walking movements that are slower, stiffer and less efficient (i.e. increased muscular activation) [12]. However, in the present research, compared to individuals with lower levels of trait conscious movement processing, higher levels of trait processing in conjunction with better inhibitory function were unexpectedly associated with higher velocity, longer steps and lower double limb support; gait patterns indicative of more effective and efficient motor output (as illustrated in Figure 1). While these results were unexpected, we speculate that these individuals were better suited to flexibly deploy an *optimum* level of conscious movement processing to meet the task requirements and more effectively adapt their gait in response to the obstacles. Research highlights that the level of conscious processing required to maintain postural stability increases with age [26]. Problems are thus likely to arise when the level of conscious processing exceeds—or falls below—what is required for successful task performance.

The methods for evaluating trait conscious movement processing (MSRS questionnaire) and short-latency inhibitory capacity (ReacStick ‘off’ accuracy) described are time-efficient, relatively inexpensive and readily portable, allowing their use in clinical environments. As patterns of cautious gait are linked to increased fall risk [3], modifying these behaviours will be of clinical importance. While the ReacStick primarily assesses prepotent motor inhibition (i.e. the ability to rapidly inhibit a dominant behavioural response), successful performance also requires resistance to distractor interference (as the performer has to attend to the light illumination status while ignoring the distraction of the stick falling). Consequently, our results could be explained by the rapid suppression of either the distracting cognitive process which leads to altered behaviour (i.e. conscious movement processing), or the behaviour itself (i.e. conscious movement processing occurs, but the associated suboptimal motor response is inhibited). Enhancing short-latency inhibition (as measured by the ReacStick) is likely to have numerous clinical benefits [21, 23]. Not only may doing so allow for better inhibition of (excessive) conscious movement processing, but also allow individuals to more effectively inhibit both external/internal distractions and (inappropriate) subcortically mediated gait patterns when making a rapid protective stepping response to avoid a fall [21, 23]. Research highlights the potential clinical efficacy of computer-based inhibition training for older adults [27]. Current evidence from this domain is encouraging, as the benefits of inhibition training in older adults appear to be maintained at 3-year follow-up [27].

## Conclusions

The data we report suggest that the combination of excessive movement processing tendencies and poor short-latency inhibitory capacity are associated with dysfunctional or ‘overly cautious’ gait. These results remained when controlling for functional balance and general cognitive function. As patterns of cautious gait are reliably linked to increased fall risk [3], modifying these behaviours are of clinical importance. The data we report suggest that clinical improvement of either excessive movement processing tendencies or short-latency inhibitory capacity may allow for improved gait and subsequently reduced fall risk.

## Data Availability

The data that support the findings of this study are available from the corresponding author, upon reasonable request.

## Footnotes

1 In the absence of any research exploring the specific influence of an interaction between inhibition and conscious movement processing on walking behaviour, this power calculation was based on research describing associations between inhibition (assessed in the same manner as in the present work) and gait behaviour in older adults [23].
